# Fenofibrate in primary sclerosing cholangitis; a randomized, double‐blind, placebo‐controlled trial

**DOI:** 10.1002/prp2.984

**Published:** 2022-07-13

**Authors:** Behzad Hatami, Mozhde Mosala, Amir Hossein Hassani, Mohammad Javad Ehsani Ardakani, Samira Gholami, Mohammad Reza Zali

**Affiliations:** ^1^ Gastroenterology and Liver Diseases Research Center, Research Institute for Gastroenterology and Liver Diseases Shahid Beheshti University of Medical Sciences Tehran Iran

**Keywords:** alkaline phosphatase, cholangitis, cholestasis, fenofibrate, liver failure, PPAR alpha, sclerosing

## Abstract

Primary sclerosing cholangitis (PSC) is a chronic cholestatic liver disease with no medical treatment proven to improve survival and postpone liver transplantation. Previous studies have shown the effectiveness of fibrates in primary biliary cholangitis. The current study prospectively evaluated the effect of fenofibrate on PSC patients. We administered 200 mg of fenofibrate to PSC patients in the intervention arm and a placebo in the control arm once per day for 6 months and evaluated liver biochemistries (alkaline phosphatase, aspartate aminotransferase, alanine aminotransferase, bilirubin, and albumin) and the Mayo Risk Score at the start and end of the study. The primary endpoint was defined as a reduction greater than 50% or normalization of ALP levels. Secondary endpoints were an improvement in the Mayo Risk Score and serum bilirubin levels. Thirty patients were included (19 female, 11 male, 40.2 ± 9.2 years old), all under treatment with Ursodeoxycholic acid prior to this study. ALP and ALT levels significantly decreased in the fenofibrate group, by 64.7% (mean difference = 557, *p* = 0.004, 95% CI = 208.72, 905.27) and 52.78%, (*p* = 0.006), respectively. The primary endpoint was achieved in 66.7% of patients (10 in 15) in the fenofibrate group versus 20% of patients (3 in 15) in the placebo group (*p* = 0.009). Other endpoints were not met. As studies have demonstrated lower levels of ALP may improve outcomes for PSC, our study resulted in significantly lower levels of ALP in the fenofibrate group, which could translate into better disease prognosis in PSC.

**Abbreviations**
ALP: alkaline phosphatase
ALT: alanine aminotransferase
AST: aspartate amonitransferase
CI: confidence interval
PBC: primary biliary cholangitis
PPARα: peroxisome proliferator‐activated receptor‐alpha
PSC: primary sclerosing cholangitis
UDCA: Ursodeoxycholic acid.

## 1 INTRODUCTION

Primary sclerosing cholangitis (PSC) is a chronic progressive cholestatic liver disease of obscure etiology characterized by inflammation, fibrosis, and stricture of intrahepatic and extrahepatic bile ducts.^1^, ^2^

Up to this date, there are no satisfactory medical treatments that will slow the progression of the disease, improve survival or delay the need for liver transplantation, which is currently the treatment of choice for patients with advanced PSC.^3^

Ursodeoxycholic acid (UDCA) is the most studied and administered drug for PSC. Although studies have suggested an improvement in liver biochemistry, it has not shown to be effective regarding liver histology, disease progression, symptom management, critical endpoints for this disease, improving survival, or a delay in the need for liver transplantation.^1^, ^4^, ^5^, ^6^, ^7^, ^8^, ^9^

Another randomized controlled trial investigating high doses of UDCA was halted unfinished due to extensive adverse events on the side of patients randomly assigned to the UDCA group compared to the placebo group, increasing the doubt on whether this medication should be used.^10^

As of 2009, the European Association for the Study of the Liver (EASL) stated that a recommendation for the general use of UDCA in PSC was not possible due to the limited data. Similarly, the guideline issued by the American Association for the Study of Liver Diseases (AASLD) in 2010 recommended against the use of UDCA for PSC patients. However, on the guideline published by the American College of Gastroenterology (ACG) in 2015, it is only recommended not to exceed doses above 28 mg/kg/day.^3^, ^11^, ^12^

As mentioned in a study performed by Ghonem et al., peroxisome proliferator‐activated receptor‐alpha (PPARα) agonists such as fenofibrate enhance cholestatic liver disease by a combination of ways, such as the transcriptional activation of the MDR3 gene, suggesting a possible alternative approach to PSC.^13^

Numerous studies have demonstrated that fibrates improve liver biochemistries in primary biliary cholangitis (PBC) patients, which was confirmed by a randomized controlled trial of bezafibrate in PBC patients with incomplete response to UDCA treatment conducted by Corpechot C et al.^14^ Given the promising results of these trials and support of molecular studies, various endeavors have been made to elucidate the effect of such treatment in PSC patients.^15^, ^16^, ^17^, ^18^

In this paper, we aimed to assess the effect of fenofibrate on several parameters and answer the following questions: Can fenofibrate cause a significant decrease in serum ALP levels? Can it diminish the Mayo risk score? Can it reduce serum bilirubin levels?

Owning to the assorted limitations of previous studies being either uncontrolled, retrospective, or suffering from a small study population, we conducted a randomized, double‐blind, placebo‐controlled trial to evaluate the effectiveness of fenofibrate in patients with PSC prospectively.

## 2 METHODS

### 2.1 Trial design and oversight

This randomized, double‐blind, placebo‐controlled trial was conducted at the Taleghani Hospital, hepatology clinic, Tehran, Iran, to investigate fenofibrate as a possible therapeutic intervention in patients with PSC. The study was conducted for 6 months, from June 2020 through November 2020, after the cessation of recruitment.

The drug and molecular target nomenclature (e.g. receptors and ion channels) in this study conforms to the IUPHAR/BPS Guide to PHARMACOLOGY nomenclature classification.^19^

The study protocol complies with the ethical guidelines of the 1975 Declaration of Helsinki. The trial was approved by the ethics committee at Taleghani hospital of Shahid Beheshti medical university, Tehran, Iran, and was registered at https://en.irct.ir (trial code: IRCT20200427047225N1). All the patients provided written informed consent.

The third author prepared the first draft of the manuscript, and it was reviewed and edited by all authors.

No medical writers or editors were involved in the development of the manuscript. The authors' decision was unanimous regarding submitting the manuscript for publication and vouch for the accuracy and completeness of the data.

### 2.2 Patients

From May to July 2020, we offered PSC patients followed up by the Taleghani Hospital liver clinic, registered from 2015 to 2020, to participate in the trial and subsequently enrolled them for screening procedures. All patients were under treatment with UDCA 13–15 mg/kg/day.

The eligibility criterion was an established diagnosis of PSC, which consists of cholestatic liver disease for at least 6 months, serum alkaline phosphatase levels 1.5 times the normal upper limit, and evidence of dilation and multifocal stricture of intrahepatic bile ducts, extrahepatic bile ducts, or both on magnetic resonance cholangiopancreatography (MRCP) or a liver biopsy supportive of this diagnosis.^12^

Exclusion criteria were as follows; advanced‐stage cancer or cardio‐pulmonary disease with a life expectancy of less than 2 years; inflammatory bowel disease requiring treatment within 3 months of study initiation (except maintenance treatment with 5‐ASA); anticipated liver transplantation within 2 years (2‐year survival less than 80% according to the Mayo Risk Score); portal hypertension complication such as esophageal variceal hemorrhage, ascites, and hepatic encephalopathy; pregnancy and nursing mothers; age under 18 or above 75 years old; co‐existing liver diseases such as alcoholic liver disease and NAFLD, autoimmune hepatitis, chronic B or C hepatitis, PBC, hemochromatosis, Wilson's disease, congenital biliary disease, and cholangiocarcinoma; preexisting cholelithiasis in bile ducts, biliary tract operations, cholecystectomy and biliary drainage procedures prior to a diagnosis of PSC; recurrent ascending cholangitis requiring hospital admission more than twice a year; hypersensitivity to fenofibrate or a history of severe side effects; acute or chronic renal failure; patients who did not provide consent.

Provided that all of the patients participating in the study had been having regular visits to our hepatology clinic, the offer of participation was made to patients who had a relatively stable course of disease and liver chemistry values.

### 2.3 Trial procedure

Patients were notified of the off‐label use of this medication and its beneficial and adverse effects. The treatment process was free of charge for the patients. After taking a disease history and performing a physical examination, blood samples were collected and sent for laboratory evaluation, measuring liver function tests (AST and ALT), alkaline phosphatase (ALP), total and direct bilirubin, and albumin levels. Baseline creatinine (Cr) and creatine phosphokinase (CPK) levels were also measured should any adverse events such as renal injury or rhabdomyolysis occur.

These tests were also performed at the end of the study at week 24. The Mayo Risk Score was also measured before and after the treatment period.

After initial evaluations, patients were randomly assigned in a 1:1 ratio to receive either placebo or fenofibrate (fenofibrate 200 mg Capsule) in identical form and packaging, to be taken after dinner once daily, for 6 months. To evaluate potential adverse effects, we checked patients' creatinine and CPK levels at baseline and every 3 months to screen and prevent possible renal injury or rhabdomyolysis, respectively.^20^ We also instructed the patients to fill out a symptom diary and contact us in case of disturbing symptoms. However, none of the participants neither experienced nor reported any adverse events.

The primary and secondary endpoints were evaluated at week 24 at the end of the study.

### 2.4 Endpoints

The primary endpoint was a reduction in serum alkaline phosphatase levels at the end of the study, defined as a decrease of greater than 50% from baseline or normalization of alkaline phosphatase levels, in which we consider the treatment as successful.^21^, ^22^, ^23^, ^24^

The secondary endpoints were as follows: a decline in the Mayo Risk Score of patients, taking into account the age, variceal hemorrhage, serum albumin, aspartate aminotransferase, and total bilirubin; and a decrease in serum bilirubin levels.^21^, ^22^, ^25^

### 2.5 Sample size and statistics

In light of the disease's rarity and a near‐zero probability in spontaneous normalization of serum biomarkers, we considered a decrease greater than 50% or normalization of ALP levels as a response to therapy. A trial with 14 participants in each group would provide the study with 80% power to detect a 50% response with a two‐sided alpha of 0.05. Hence, we enrolled 30 patients to be randomized into either group while allowing for one person drop‐out per group.

Patients' laboratory characteristics are demonstrated as mean ± standard deviation or median (IQR). The Wilcoxon signed‐rank test was used to evaluate changes in laboratory values within two groups, except for alkaline phosphatase, albumin levels, and Mayo risk score. Paired T‐test was used to assess changes within groups in alkaline phosphatase, albumin levels, and the Mayo risk score.

Mann–Whitney and Independent sample T‐test were used to evaluate differences in the study parameters between the intervention and control groups at the baseline and end of the study. In the case of significant results, depending on the outcome data distribution, sensitivity analysis with the possible option of performing the corresponding nonparametric test, undertaking analysis with and without adjustment for baseline characteristics, or excluding outliers was performed. A two‐sided P‐value of less than 0.05 was considered statistically significant. The analysis was conducted with the intention to treat. Statistical analysis was performed using the IBM SPSS Statistics 26.0 package.

### 2.6 Randomization and blinding

After the eligibility screening, to ensure patients' random allocation into either group, an independent statistician assigned each patient a number based on their recruitment order. Subsequently, these numbers were inserted into the RV uniform 0.1 function in the SPSS application. In the case of an answer greater than 0.5, the patient was allocated to the intervention group, otherwise designated to the control group. The allocation sequence and randomization list were kept from the recruiters, investigators, and study analyst for the trial's whole duration, by the independent statistician.

According to a sheet the independent statistician had provided him, a staff member not involved in the treatment procedure and outcome assessment filled out medication containers and numbered them, each corresponding to a patient's recruitment number. Afterward, the investigators provided patients with the containers which matched their number, hence masking both patients and investigators to the allocated group.

### 2.7 Nomenclature of targets and ligands

Key protein targets and ligands in this article are hyperlinked to corresponding entries in http://www.guidetopharmacology.org, the common portal for data from the IUPHAR/BPS Guide to PHARMACOLOGY (Harding et al.), and are permanently archived in the Concise Guide to PHARMACOLOGY 2019/20 (Alexander et al.).^19^, ^26^, ^27^

## 3 RESULTS

### 3.1 Patients

**FIGURE 1:**
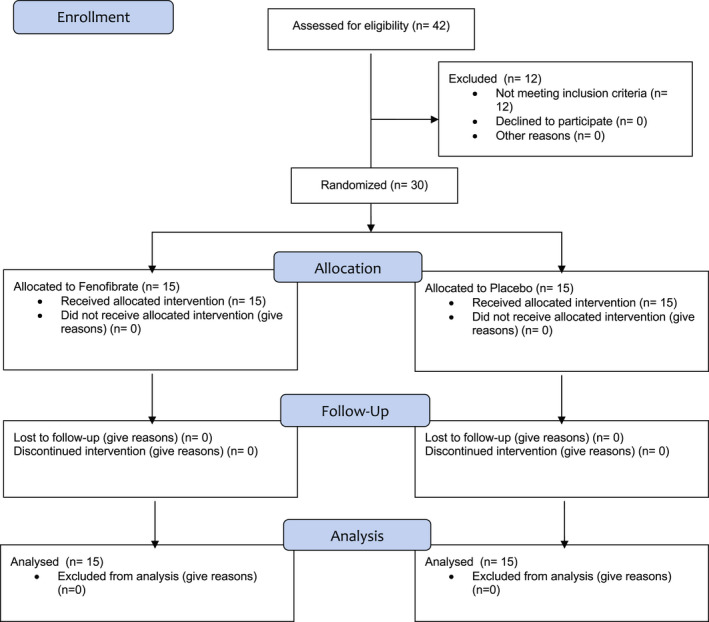
Enrollment and trial procedure.

Thirty patients were enrolled (19 female, 11 male, 40.2 ± 9.2 years old). All patients completed the study (Figure 1). Fenofibrate was well tolerated across both groups, and none of the patients reported any adverse events. Renal biochemistry and muscle enzymes remained stable during treatment, and no patient experienced renal or muscle injury. Furthermore, none of the participants received biliary interventions, antibiotics, or concomitant medication changes during the study, potentially impacting biochemical values under investigation. The baseline characteristics of patients did not differ between the two groups (Table 1).

**TABLE 1:** Demographics and baseline characteristics

Characteristics^a^	Fenofibrate (*N* = 15)	Placebo (*N* = 15)	*p* value
Age, (year)	40.1 ± 10.2	40.3 ± 8.4	0.969*
Gender, Female: Male	11:4	8:7	0.256**
Alkaline phosphatase, (U/L)	859.7 ± 822.9	622.0 ± 489.7	0.548***
Aspartate aminotransferase, (U/L)	81.1 ± 66.9	68.5 ± 70.9	0.547***
Alanine aminotransferase, (U/L)	124.1 ± 127.7	74.2 ± 79.4	0.158***
Bilirubin, Total (mg/dL)	2.6 ± 4.5	1.8 ± 1.7	0.361***
Bilirubin, Direct (mg/dL)	1.5 ± 2.9	0.9 ± 1.1	0.381***
Albumin, (g/dL)	4.2 ± 0.5	4.3 ± 0.3	0.414*
Mayo Risk Score^b^	0 ± 1.1	−0.2 ± 0.9	0.667*
Inflammatory bowel disease	2	3	0.620**

*Note*: Data are demonstrated as means ± SD.

^a^ The normal range for alkaline phosphatase is up to 306 U per liter; for aspartate aminotransferase and alanine aminotransferase, up to 40 U per liter; for bilirubin, up to 1.2 mg per deciliter; and for albumin, 3.5–5.0 g per deciliter. To convert bilirubin values to micromoles per liter, multiply by 17.1.

^b^ The Mayo Risk Score is calculated as demonstrated: (0.0295 * [age in years]) + (0.5373 * LN [total bilirubin in mg/dL]) ‐ (0.8389 * [serum albumin in g/dL]) + (0.5380 * LN (AST in IU/L) + (1.2426 * [points for variceal bleeding]); a score equal to 0 or less is considered low risk, greater than 0 but less than 2 is intermediate risk, and scores greater than 2 are associated with high risk.^25^

*p‐value reported based on independent sample *t*‐test, **p‐value based on chi‐square test, ***p‐value based on Mann–Whitney test.

### 3.2 Endpoints

Liver biochemistries, serum alkaline phosphatase, albumin, and bilirubin levels obtained at baseline and the end of the study are demonstrated in Table 2.

**TABLE 2:** Endpoint comparison of laboratory findings at baseline (day 0) and at 24 weeks for placebo and fenofibrate group

Serum biomarkers	Group				*p*‐value
	Placebo		Fenofibrate		
	Mean (SD)	Median (IQR)	Mean (SD)	Median (IQR)	
Aspartate Aminotransferase (U/L) baseline	68.5 (70.9)	39.0 (30.0, 70.0)	81.1 (66.9)	51.0 (28.0, 110.0)	
Aspartate Aminotransferase (U/L) 24 weeks	53.7 (37.7)	45.0 (24.0, 81.0)	49.4 (35.8)	36.0 (26.0, 57.0)	0.884^a,^***
*p*‐value^c,^**	0.875		0.069		
Alanine Aminotransferase (U/L) baseline	74.2 (79.4)	53.0 (28.0, 68.0)	124.1 (127.7)	70.0 (41.0, 186.0)	
Alanine Aminotransferase (U/L) 24 weeks	52.5 (34.3)	43.0 (22.0, 84.0)	58.6 (43.9)	44.0 (31.0, 77.0)	0.674^b,^***
*p*‐value^c,^**	0.532		0.006*		
Bilirubin Total (mg/dL) baseline	1.8 (1.7)	1.4 (0.7, 2.0)	2.6 (4.5)	0.9 (0.6, 2.3)	
Bilirubin Total (mg/dL) 24 weeks	1.8 (1.8)	1.0 (0.7, 2.3)	1.3 (1.4)	0.8 (0.5, 1.1)	0.318^a,^***
*p*‐value^c,^**	0.955		0.234		
Bilirubin direct (mg/dL) baseline	0.9 (1.1)	0.5 (0.3, 1.1)	1.5 (2.9)	0.2 (0.2, 1.4)	
Bilirubin direct (mg/dL) 24 weeks	1.0 (1.4)	0.3	0.7 (1.0)	0.3 (0.2, 0.6)	0.511^b,^***
*p*‐value^c,^**	0.562		1.00		
Alkaline Phosphatase (U/L) baseline	622.0 (489.7)	501.0 (268.0, 799.0)	859.7 (822.9)	590.0 (286.0, 1160.0)	
Alkaline phosphatase (U/L) 24 weeks	557.8 (337.1)	500.0 (333.0, 645.0)	302.7 (272.9)	225.0 (150.0, 297.0)	0.002^**a**,^*^,^***
*p*‐value^d,^**	0.649		0.004*		
Albumin (g/dL) baseline	4.3 (0.3)	4.2 (4.0, 4.6)	4.2 (0.5)	4.0 (3.8, 4.5)	
Albumin (g/dL) 24 weeks	4.2 (0.5)	4.0 (3.9, 4.6)	4.1 (0.3)	4.0 (3.9, 4.5)	0.619^b,^***
*p*‐value^d,^**	0.305		0.763		
MAYO risk score baseline	−0.2 (0.9)	−0.1 (−1.2, 0.8)	0.0 (1.1)	−0.2 (−1.1, 0.8)	
MAYO risk score 24 weeks	−0.2 (1.1)	−0.3 (−0.9, 0.7)	−0.3 (0.8)	−0.5 (−0.8, 0.3)	0.617^b,^***
*p*‐value^d,^**	0.960		0.211		

*Note*: Statistical method implemented: ^a^Mann–Whitney, ^b^Independent sample T test, ^c^Wilcoxon, ^d^Paired T test.

*Imply statistical significance, **p‐value comparing baseline with 24 week lab value, ***p‐value comparing placebo with fenofibrate treatment at 24 week.

In the fenofibrate group serum alkaline phosphatase at 24 weeks (mean = 302.7, SD = 272.9 U/L, median = 225) was significantly decreased compared to baseline (mean = 859.7, SD = 822.9 U/L, median = 590, *t*[14] = −3.43, *p* = 0.004, 95% CI = 208.72, 905.27). In contrast, no significant change in alkaline phosphatase was observed in the placebo group (*p* = 0.64). This difference remained significant after sensitivity analysis with applying nonparametric tests. Moreover, 66.7% (10 of 15) of the patients in the fenofibrate group versus 20% (3 of 15) of the placebo group reached the primary endpoint (*χ* ^2^ = 6.65, *p* = 0.009). At 24 weeks the alkaline phosphatase level was significantly lower in the fenofibrate group (mean = 302.7, SD = 272.9 U/L, median = 225) compared to the placebo group (mean = 557.8, SD = 337.1, median = 500), (*z* = −3.09, *p* = 0.002). After sensitivity analysis, the results remained unchanged, excluding outliers, and performing an ANCOVA test with adjustment for gender and baseline alkaline phosphatase level.

The result of the Wilcoxon rank test indicated that there was a significant difference in alanine aminotransferase levels between baseline (mean = 124.1, *SD* = 127.7, median = 70) and after 24 weeks of treatment (mean = 58.6, *SD* = 43.9, median = 44) in only fenofibrate group (z = −2.72, *p* = 0.006). In contrast, no improvement in alanine aminotransferase levels was reported in the placebo group (0.53). Fenofibrate was not associated with a statistically significant decrease in serum aspartate aminotransferase, bilirubin, and albumin levels (*p* > 0.05). Moreover, fenofibrate did not lead to a significant improvement in the Mayo Risk Score (*p* = 0.21).

## 4 DISCUSSION

PSC is a chronic cholestatic liver disease with unknown etiologies, although some studies have suggested genetic and immunologic factors to be at play.^1^, ^2^ With an incidence rate of approximately 0.77 per 100 000 person‐years,^28^ PSC is considered rare; nonetheless, it is a troublesome disorder due to the lack of curative treatment or even medical therapy. Patients may follow a progressive deterioration resulting in portal hypertension and liver failure, which is eventually managed by liver transplantation in the advanced stages of the disease. Apart from cholestatic symptoms and complications, PSC is often associated with inflammatory bowel disease (IBD).^1^, ^2^ PSC has been shown to increase the risk of cholangiocarcinoma.^1^ When accompanied by IBD, it has been demonstrated to be associated with a higher risk of colorectal cancer.^29^ With controversies regarding UDCA's use and its ineffectiveness on the disease course, new means of treatment have emerged.

In the study conducted by Day AP et al., bezafibrate was shown to be effective in lowering alkaline phosphatase levels in patients treated for hyperlipidemia.^30^ Thereafter, several studies have suggested using fibrates for cholestatic liver disease,^13^, ^31^ which has been proven beneficial in primary biliary cholangitis (PBC).^14^ The first instance of implementing this therapy on PSC patients was reported by Kita et al.,^32^ followed by multiple other studies demonstrating the effectiveness of fibrates, mostly bezafibrate,^15^, ^16^, ^33^ but also fenofibrate.^17^, ^18^ In almost all cases, they reported a significant decline in ALP, and some reported a decline in AST, ALT, or both.^15^, ^16^, ^17^, ^18^, ^32^, ^33^

Peroxisome proliferator‐activated receptors (PPARα) belong to the nuclear hormone receptor superfamily, which are ligand‐activated transcription factors. The three PPAR isoforms are α, β/δ, and γ. PPARα takes part in cholesterol and bile acid homeostasis. It downregulates bile acid synthesis and is believed to be mediated through the multidrug resistance protein 3 (MDR3). MDR3 has an essential role in bile salt secretion, and its gene mutation has been shown to result in cholestasis and can lead to various liver disorders. Thus, PPAR alpha and MDR3 are crucial pharmacological targets for this disease. Despite evidence of the efficacy of fibrates in both animal and clinical studies, the mechanism by which fibrates decrease biochemical markers of cholestasis remains unclear. The proposed mechanism for this observation is the trans‐activation of the MDR3 gene by the PPAR receptor (mainly the PPAR‐α isoform) on hepatocytes. The activation of the PPAR‐α receptor and the expression of the MDR3 gene result in the regulation of bile acid synthesis and secretion, respectively, possibly causing the anti‐cholestatic effects of fibrates.^13^ Moreover, additional effects of the activation of PPAR‐α have been postulated to aid the mitigation of hepatic cholestatic disease, although yet undetermined. These effects include the upregulation of MRP3 and MRP4 efflux transporters, association with anti‐inflammatory regulation, and indirectly interceded Nuclear Farnesoid X Receptor (FXR) effects.^13^

However, bezafibrate is not FDA approved, and it is a nonspecific activator of the PPAR receptor, activating all three alpha, beta‐delta, and gamma isoforms at the same molar concentrations. In contrast, fenofibrate, an FDA‐approved medication with a 10‐fold selectivity for PPAR alpha vs. gamma, specifically activates the PPAR‐α isoenzyme,^34^ which directly upregulates MDR3 expression, thus eliminating the need of bezafibrate's super physiological doses to achieve the same effects.

Nevertheless, data on the effectiveness of fibrates on PSC patients are scarce, and discrepancies exist in the literature, primarily due to the small study population, lack of randomized placebo‐controlled studies, and the use of bezafibrate as the intervention agent.

In the current study, we investigated the effect of fenofibrate on PSC patients by conducting a prospective, randomized, double‐blind, placebo‐controlled trial.

Our study demonstrates that fenofibrate can significantly reduce alkaline phosphatase levels by 64.79%, which multiple studies have proposed this reduction in ALP to be associated with better disease prognosis and a reliable surrogate marker to evaluate clinical outcomes in PSC patients.^23^, ^24^, ^35^, ^36^

Our study's finding is in keeping with previous studies mentioned investigating fibrates on PSC patients^15^, ^16^, ^17^, ^18^, ^32^, ^33^ and studies performed on PBC patients^14^, ^31^ suggesting fenofibrate as a potentially effective pharmacologic treatment for managing cholestasis in PSC due to the favorable prognostic factor.

Moreover, fenofibrate significantly decreased alanine aminotransferase levels by 52.78%, suggesting an amelioration in hepatic parenchymal inflammation and function.

On the other hand, we found fenofibrate ineffective in improving aspartate aminotransferase and serum bilirubin levels. In contrast to ALP, a reduction in these parameters, including alanine aminotransferase, was not unanimous across studies and calls for further investigation.

With the unaffected laboratory values taken into account, we observed that fenofibrate did not ameliorate the Mayo Risk Score, raising the question of whether fenofibrate can improve the outcome in PSC patients, as studies have suggested being associated with the decrease in alkaline phosphatase.^23^, ^24^, ^35^, ^36^ We assume this might primarily be due to the absence of ALP in the Mayo Risk Score as it is only starting to be recognized as a potential new parameter to predict disease outcome in PSC. Moreover, our study's relatively short duration undermines our ability to assess the effect of fenofibrate on disease prognosis properly; therefore, future studies incorporating longer periods of treatment and follow up might be able to answer this question.

This study has some limitations. As discussed earlier, one of the limitations of our study was the concurrent use of UDCA. Owning to the lack of treatment options for PSC patients, although with limited effectiveness, almost all of them are under treatment by UDCA. Adding that to the rarity of the disease, it is an arduous task to find PSC patients who are not consuming UDCA. Thus, yet a relatively small study population was another limitation of this study. The results need to be considered with caution since this was just a small single‐center trial, and a larger study population from more sites or even different countries may lead to other outcomes. The short time of fenofibrate administration (6 months) was another limitation to the study and diminished our ability to establish a well‐grounded response in patients and observe the full scope of fenofibrate's impact on the study endpoints and patients' prognosis. Unfortunately, given the recent Covid‐19 outbreak and the particular situation evolving around the matter, we were not able to follow up with the participants in the study. Alas, it would have provided us with a great deal of information concerning the rebound of biomarkers following discontinuation of fenofibrate. Another impediment was the lack of histopathologic assessment of the liver tissue and only evaluating laboratory parameters' improvement, which might not directly translate into clinical benefits. Nevertheless, as discussed earlier, studies have seen a positive effect on patient survival when ALP levels decrease significantly.

Considering the issues presented emphasizes the need for future trials incorporating multiple features to make an effort to surpass the limitations of the current study.

Desirable endeavors toward the latter would be but not limited to a larger study population, risk stratification, and disease grading at entry, longer duration of intervention and follow up, more frequent assessment of laboratory values, investigation of other aspects of the disease by incorporating imaging techniques such as elastography, bile sample analysis, histology, and validated endpoints according to the literature in order to assess clinically significant outcomes.^21^, ^22^

Also, given that the small numbers and variability make an assessment of causality difficult, it might be informative to correlate the observed effects on biochemical markers with some measure of pharmacology, such as exposure of fenofibrate in the plasma or bile, or with a marker of fibrate target engagement such as lipid changes.

## 5 CONCLUSIONS

In conclusion, fenofibrate can significantly lower ALP and ALT levels in PSC patients and demands further investigation through more extensive studies to establish a reliable response, evaluate multiple aspects of its effect, and assess the intervention's prognostic value.

## AUTHOR CONTRIBUTION

Hatami B and Zali MR designed the study. Mosala M, Hatami B, Hassani AH, and Ehsani Ardakani MJ recruited patients and conducted the trial. Hatami B, Mosala M, Ehsani Ardakani MJ, and Gholami S undertook data collection. Mosala M and Gholami S performed the statistical analysis and interpreted the data. Hassani AH and Hatami B drafted the manuscript, and all authors took part in the critical revision and editing process.

## FUNDING INFORMATION

This study did not receive any grants and was not funded by any organization.

## DISCLOSURE

The authors of this study declare no conflict of interest.

## ETHICS

The study protocol complies with the ethical guidelines of the 1975 Declaration of Helsinki. The trial was approved by the ethics committee at Taleghani hospital of Shahid Beheshti medical university, Tehran, Iran.

The trial was registered at https://en.irct.ir (IR.SBMU.MSP.REC.1398.198).

## CONSENT TO PARTICIPATE AND CONSENT FOR PUBLICATION

All the patients provided written informed consent to participate in this study and its publishment.

## Data Availability

The datasets generated and analyzed during the current study are available from the corresponding author upon request.
